# Human toxocariasis and atopy

**DOI:** 10.1051/parasite/2020029

**Published:** 2020-05-13

**Authors:** Jean-François Magnaval, Judith Fillaux, Sophie Cassaing, Alexis Valentin, Xavier Iriart, Antoine Berry

**Affiliations:** 1 Service de Parasitologie Médicale, Faculté de Médecine, Université de Toulouse 31000 Toulouse France; 2 Service de Parasitologie et Mycologie, Université de Toulouse, Centre Hospitalier Universitaire de Toulouse TSA 40031-31059 Toulouse cedex 9 France; 3 PharmaDev, Faculté de Pharmacie, Université de Toulouse, IRD, UPS 31062 Toulouse cedex 9 France; 4 Centre de Physiopathologie Toulouse-Purpan (CPTP), Université de Toulouse, INSERM, CNRS, UPS TSA 40031-31059 Toulouse cedex 9 France

**Keywords:** Human toxocariasis, Atopy, Outcome, Clinical picture, Eosinophilia, IgE

## Abstract

To assess the possible influence of atopy on the clinical picture of human toxocariasis, a retrospective study was carried out using file records for patients who attended the Outpatient Clinic of Parasitology in Toulouse University Hospitals. A total of 106 file records for patients who had been diagnosed with common/covert toxocariasis were extracted from the database. Forty-nine patients (20 females and 29 males) were considered atopic since they exhibited a long (≥ 1 year) history of various allergic issues along with a titer ≥ 0.7 kIU/L for specific IgE against at least two out of nine mixes of common inhalant allergens. Fifty-seven patients (42 females and 15 males) were designated nonatopic on the basis of a negative result (<0.35 kIU/L) of the test for specific IgE. Demographic (age and sex), clinical (20 signs or symptoms) and laboratory (blood eosinophil count, eosinophil cationic protein, serum total IgE, and specific anti-*Toxocara* IgE) variables were investigated by bivariate analysis followed by multivariate regression analysis using “atopy” as the outcome variable. On the basis of our results, the clinical or laboratory picture of toxocaral disease was not affected by the presence of an atopic status.

## Abstract (fr)

Pour évaluer la possible influence de l’atopie sur la présentation clinico-biologique de la toxocarose humaine, une étude rétrospective a été réalisée à partir des dossiers de patients vus à la Consultation du Service de Parasitologie-Mycologie du CHU de Toulouse. Cent-six dossiers de patients diagnostiqués comme ayant la forme commune de la toxocarose ont été extraits de la base de données. Quarante-neuf patients (20 femmes et 29 hommes) ont été considérés comme atopiques, eu égard à une longue (≥ 1 an) histoire de manifestations allergiques couplée à une recherche positive (≥ 0.7 kUI/L) des IgE spécifiques contre au moins deux parmi 9 mélanges de pneumallergènes communs. Cinquante-sept patients (42 femmes et 15 hommes) ont été classés non atopiques sur la base d’un résultat négatif (< 0.35 kUI/L) de la recherche d’IgE spécifiques. Les variables démographiques (âge et sexe), cliniques (20 signes ou symptômes) et biologiques (numération des éosinophiles sanguins, dosage des protéines cationiques des éosinophiles, des IgE totales et des IgE spécifiques anti-*Toxocara*) ont été l’objet d’une analyse statistique bivariée suivie par une régression logistique multivariée, en utilisant “atopie” comme variable à expliquer. Selon nos résultats, le tableau clinique et biologique de la toxocarose n’est pas modifié par la présence d’un état atopique.

## Introduction

Human toxocariasis is a worldwide zoonotic helminthiasis due to infection with the larvae of *Toxocara cani*s or *Toxocara cati*, roundworms that parasitize canids or felids, respectively [15]. Adult stages of these ascarid helminths dwell in the upper digestive tract of the definitive hosts. Eggs passed in the feces must be in the soil to become embryonated and infective. Most frequently, humans become infected by ingesting embryonated eggs present in nearby soil or on raw vegetables [12, 34]. In the duodenum, larvae hatch from embryonated eggs and then migrate through the body. Larvae continuously release soluble glycoprotein antigens of excretory-secretory origin that contain at least one potent allergenic fraction [36]. Toxocaral infection results in several disorders that can be classified either as systemic (generalized) – including major visceral larva migrans (VLM) syndrome and covert/common toxocariasis – or compartmentalized when the disorders are associated with ocular or neurological involvement. Various allergic issues are commonly observed during the course of systemic syndromes [15, 24].

Atopy and allergy are different paradigms. Atopy is the predisposition to produce high levels of specific IgE antibodies against common environmental or food allergens and subsequently develop immediate hypersensitivity in response to exposure to these substances [38]. Allergy is a more or less harmful immune-mediated inflammatory response to environmental substances known as allergens, which may induce allergic diseases such as allergic asthma, allergic rhinitis, atopic dermatitis, or food allergy [4]. Therefore, atopy refers to genomics, whereas allergy defines a phenotype. However, allergic asthma represents a peculiar situation among allergy-related diseases. Asthma is a heterogeneous condition due to chronic inflammation of the lower respiratory tract. This key feature is linked to bronchial hyperresponsiveness (BHR) that arises from complex not fully understood gene-environment interactions [29, 31]. Consequently, it should be emphasized that asthma may develop in the absence of any allergy.

In 1994, a first article reported an elevated seroprevalence of toxocariasis in young schoolchildren with allergic asthma [8]. Since then, there have been many studies investigating the relationship between this zoonosis and allergy or atopy, including two meta-analyses concerning either allergic asthma [3] or skin allergy [30]. They were carried out in the general population, thus classifying the patients as atopic/allergic or nonatopic/nonallergic, and then searching for anti-*Toxocara* antibodies or following Buijs et al.’s study design [8], namely, determining toxocariasis seroprevalence in atopic or allergic patients. The results varied, but a trend emerged from these surveys, suggesting that toxocariasis showed an increased prevalence in atopic patients. However, none of these studies investigated the possible modulation by atopy of the clinical and laboratory picture of toxocaral disease. Only one prospective survey in Spain partially investigated the clinical and laboratory expression of toxocariasis in atopic *vs.* nonatopic subjects. Both groups were formed only on the basis of a positive result for toxocariasis serology [13]. Despite this lack of knowledge about the pathophysiology of this zoonosis, one can find medical educational material on the internet stating that patients with atopy may experience toxocariasis with increased severity [28].

Therefore, the aim of the present retrospective study was to compare the clinical and laboratory pictures of active common/covert toxocariasis in atopic and nonatopic patients.

## Patients, materials and methods

### Ethical considerations

The solicited local Ethics Committee (“*Comité pour la Protection des Personnes du Sud-Ouest et Outre-mer II*”) declared that retrospective studies that analyzed previous file records did not require any approval when all results were anonymous and no further diagnostic tests were carried out. Because our investigation procedure was simply standard implementation of known clinical practices with the guidance of existing diagnostic tools, only oral informed consent was required from the patients at the time of their attendance at the Clinic of Parasitology. All diagnostic investigations were performed in accordance with the Declaration of Helsinki.

### Patients

At the Outpatient Clinic of the Department of Parasitology and Mycology at Toulouse University Hospitals, France, the diagnosis of active common/covert toxocaral disease was made at the end of a meticulous protocol that has been detailed elsewhere [25]. Patients presenting with only ocular or neurological toxocariasis were not considered since the compartmentalized forms of this zoonosis result from a pathogenic process that differs from that of the generalized forms, and display a different clinical picture.

Briefly, for any outpatient who exhibited various clinical disorders and blood eosinophilia, a detailed questionnaire inquired about demographics (age and sex), and epidemiological data. Medical history was recorded, and special attention was paid to any previous report of signs or symptoms of allergy. The time interval between the onset of manifestations and attendance at the clinic was recorded in months. The clinical picture was evaluated on general examination, and plain chest radiography and abdominal ultrasound were prescribed. The panel of laboratory investigations included a variety of tests that aimed to determine the cause of eosinophilia, including the serological test for toxocaral etiology. Importantly, the clinical and laboratory picture of the most frequent form of this zoonosis, namely, common/covert toxocariasis, is not specific [21, 24]. Moreover, many subjects have residual anti-*Toxocara* antibodies due to past self-cured infections. Therefore, the diagnosis of active common/covert toxocariasis was made by exclusion in patients exhibiting a positive result for toxocariasis serology once the protocol ruled out other causes of eosinophilia. All patients were investigated by the first author (JFM).

From our department databank, 134 file records of patients who had been clinically and serologically diagnosed as having active, symptomatic, common/covert toxocariasis were extracted. These patients did not have any active concurrent helminthiasis. A first round of selection retained file records on the basis of the following criteria: absence of any concurrent active protozoal or fungal infection; absence of any ongoing bacterial or viral infection; no past history of helminthiasis or tissue-dwelling protozoal infection; absence of commensal or pathogenic intestinal protozoa in the microscopy examination of stools; absence of any stay or repeated travel outside Western Europe; and no immigration from a country outside Western Europe.

Then, file records from patients with allergic asthma were excluded from the study to avoid any interference caused by the specific genomics of this disease (see above). Based on the combined results from the medical questionnaire and the detection of specific IgE against common inhalant allergens (see the “Laboratory Methods” section), a further screening round excluded patients displaying a history of clinical allergy but a result < 0.35 kilo International Units/Liter (kIU/L) – in the test for specific IgE. This selection step aimed to reduce the risk of false diagnosis of atopy in these helminth-infected patients.

*Toxocara canis* larval excretory-secretory antigens (TES-Ag) are rich in carbohydrates [26]. Since these IgE-binding epitopes are shared between parasites and allergens [7], IgE directed against helminth antigens may cross-react with standard allergenic extracts [6]. A subsequent selection step excluded patients without any past history of allergy but with a result for specific IgE ≥ 0.35 kIU/L, since the presence of anti-allergen IgE in nonatopic subjects is possible [1].

Finally, 106 file records were retained. Forty-nine patients (20 females and 29 males) were classified as atopic according to the criteria of the American Academy of Allergy, Asthma & Immunology [5]. They displayed a past and long-term history (≥ 1 year) of various allergic disorders, along with a result ≥ 0.7 kIU/L for the test for specific IgE against at least two mixes of common inhalant allergens.

Fifty-seven patients (42 females and 15 males) without a long-term history of allergy and displaying a result of the test for specific IgE of < 0.35 kIU/L were considered nonatopic.

**Figure 1:**
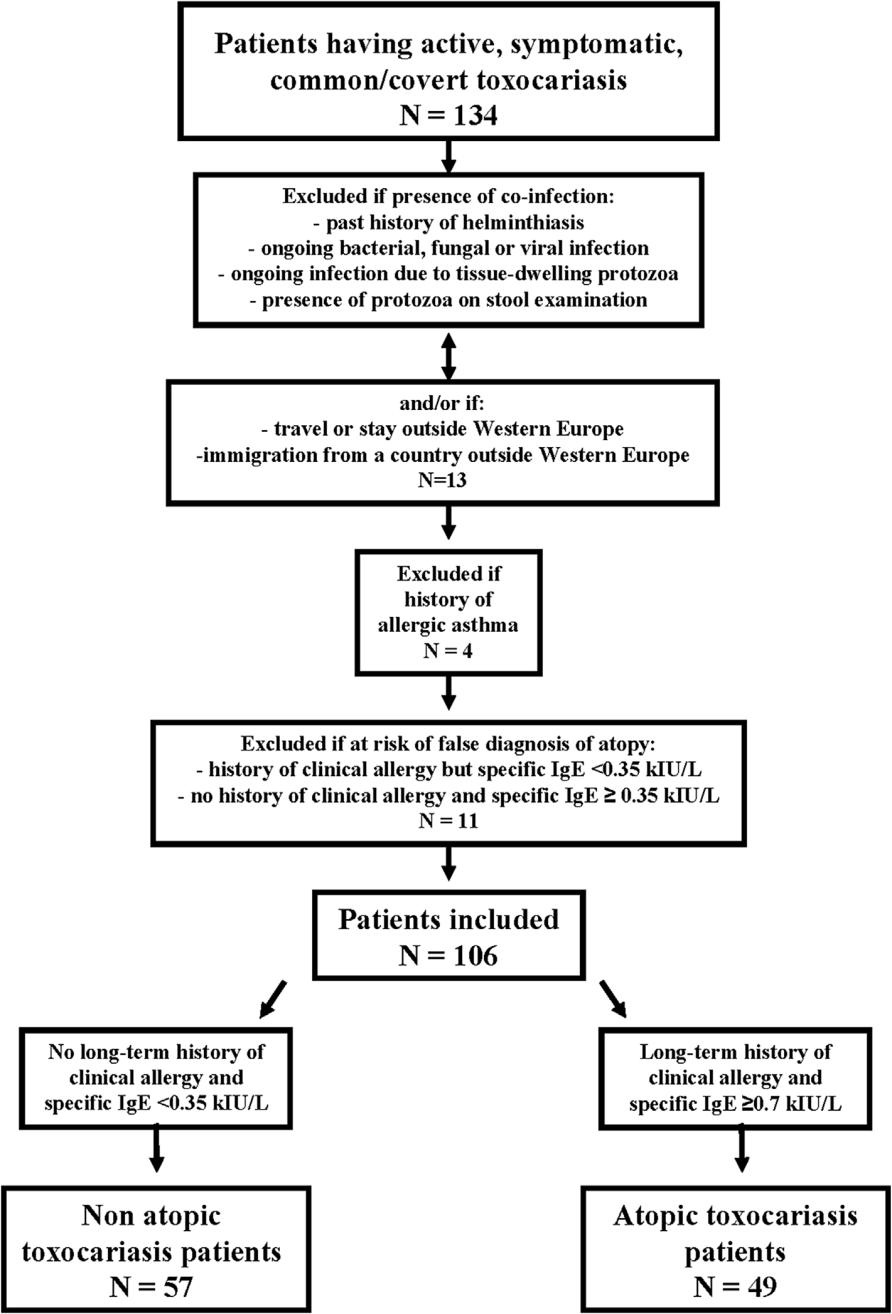
Flowchart of the selection process of 106 toxocariasis patients.

The selection process is displayed graphically as a flowchart in Figure 1.

### Laboratory methods

The immunodiagnosis of toxocariasis was based on a western blot procedure detecting specific IgG against TES-Ag. This antigenic reagent was produced in the Department of Parasitology and Mycology. The presence of a banding pattern displaying a positive reaction for the low molecular weight bands (24, 28, 30, and 35 kDa) was evidence of a specific positive result [22].

Total and differential blood counts were obtained with an SHT 330™ blood analyzer (TOA Sysmex, Roche Diagnostics, Neuilly-sur-Seine, France). Eosinophilia was expressed in G cells/L.

Assays for serum total IgE and eosinophil cationic protein (ECP) were carried out using the Phadia CAP^®^ System (now ImmunoCAP^®^, ThermoScientific, Montigny-le-Bretonneux, France). The results are expressed in kIU/L for total IgE and in μg/L for ECP. IgE against inhalant allergens was also detected on the CAP^®^ system using the following mixes: epidermal and animal proteins (ex1 and gh2), grasses (gx1 and rx1), molds (mx1), trees (tx1 and tx4) and weeds (wx1 and wx6) [37]. The results are given in kIU/L.

The detection of specific anti-TES-Ag IgE was adapted from a previously described method [23] that used TES-Ag along with Pharmacia Phast^®^ reagents in a Pharmacia Fluorocounter 96™. The results are expressed in *Toxocara* units (TU)/L.

Except for total and differential blood counts, which were performed in the Department of Hematology, all tests were carried out in the Department of Parasitology and Mycology. The laboratory procedures are submitted every two years to a quality audit by inspectors from COFRAC, the French branch of the International Accreditation Forum (http://www.iaf.nu/) for compliance with ISO standard #15189, and then accredited.

### Statistical analysis

The set of demographic, clinical and laboratory variables that were analyzed are displayed in Tables 1 and 2. Data from continuous variables were log_e_-transformed, so the means were geometric. To be included in the statistical analysis, a sign or symptom had to display at least two occurrences in at least one group of patients.

**Table 1:** Bivariate analysis of demographic and clinical categorical variables recorded in 106 toxocariasis patients.

	Nonatopic (*n* = 57)	Atopic (*n* = 49)	*p*
Variable	% (*n*)	% (*n*)	
Demographic data			
Females	73.7 (42)	40.8 (20)	
Males	26.3 (15)	59.2 (29)	<0.001*
Clinical reason for attending the clinic			
Abdominal pain	7.02 (4)	2.04 (1)	0.370 †
Arthralgia and/or myalgia	5.26 (3)	4.08 (2)	1.000 †
Chronic cough	10.53 (6)	0 (0)	0.071 †
Chronic weakness	40.35 (23)	34.69 (17)	0.549 *
Cutaneous allergy ‡ ||	14.03 (8)	10.20 (5)	0.397 †
Pruritus *sine materia*	7.02 (4)	6.12 (3)	1.000 †
Wheeze	5.26 (3)	16.33 (8)	0.107 †
Clinical data recorded during consultation			
Arthralgia and/or myalgia	19.30 (11)	26.53 (13)	0.375 *
Chronic irritative cough	31.58 (18)	16.33 (8)	0.068 *
Colic pain ||	22.81 (13)	24.49 (12)	0.839 *
Conjunctivitis ||	26.32 (15)	30.61 (15)	0.624 *
Cutaneous allergy ‡ ||	22.81 (13)	20.41 (10)	0.765 *
Facial and or hand edema ||	15.79 (9)	4.08 (2)	0.060 †
Frequent headache	17.54 (10)	12.24 (6)	0.448 *
Intermittent diarrhea	5.26 (3)	6.12 (3)	1.000 †
Otorhinolaryngeal allergy ¥	38.60 (22)	51.02 (25)	0.199 *
Paresthesia	7.02 (4)	2.04 (1)	0.370 †
Pruritus *sine materia*	33.33 (19)	18.37 (9)	0.081 *
Weakness	71.92 (41)	77.55 (38)	0.508 *
Wheeze ||	8.77 (5)	16.33 (8)	0.253 †

* Pearson’s χ^2^ test;

† Fisher’s exact test;

‡ eczema, prurigo, rash, urticaria;

|| found by clinical examination;

¥ pharyngitis, rhinitis (rhinorrhea, nasal congestion, sneezing), sinusitis.

**Table 2:** Bivariate analysis of demographic, clinical and laboratory continuous variables recorded in 106 toxocariasis patients.

Variable	Nonatopic (*n* = 57)	Atopic (*n* = 49)	*p*
Age (years)			
Mean*	46.9	32.3	
95% confidence interval	[40.5 – 54.3]	[26.7 – 39.1]	0.002†
Weight (kg)			
Mean*	64.5	61.5	
95% confidence interval	[59.6 – 69.7]	[54.7 – 69.3]	0.50†
Time Interval§ (months)			
Mean*	5.7	6.1	
95% confidence interval	[4.5 – 7.4]	[4.4 – 8.4]	0.97‡
Eosinophil count (G/L)			
Mean*	1.2	0.9	
95% confidence interval	[1.0 – 1.4]	[0.8 – 1.1]	0.02‡
ECP|| (μg/L)			
Mean*	37.8	26.6	
95% confidence interval	[30.2 – 47.5]	[21.3 – 33.2]	0.02‡
Total IgE (kIU/L)¥			
Mean*	463.8	529.4	
95% confidence interval	[284.3 – 757.5]	[377.5 – 742.4]	0.54‡
sIgE¶ (TU/L)#			
Mean*	7.1	15.4	
95% confidence interval	[4.7 – 10.8]	[7.9 – 30.3]	0.11‡

* All geometric;

† Student’s *t* test;

‡ Mann-Whitney *U* test;

§ Time interval between the onset of manifestations and attendance at the clinic;

|| eosinophil cationic protein, normal range: [0–14];

¥ kilo International Units, cut-off value: 2 kIU/L;

¶ specific IgE against excretory-secretory antigens from *T. canis* larvae;

# kilo *Toxocara* units, normal range: [0–5].

Environmental parameters, including occupation, number of pet dogs or cats at home, presence of a kitchen garden, or type of residence (urban, semi-urban or rural), were recorded and then submitted to bivariate analysis because they could influence both dependent and independent variables, thus acting as confounders.

The statistical package Intercooled Stata™ (StataCorp LLC, College Station, TX, USA) was used for statistical analysis. Bivariate analysis of the data set used Pearson’s χ^2^ and Fisher’s tests as appropriate for the categorical variables, or Mann–Whitney *U* and Student’s *t* tests as appropriate for the continuous variables.

Using “atopy” as the outcome variable, a multivariate regression analysis (MRA) was conducted. Only the variables that differed with *p* ≤ 0.15 by bivariate analysis were tested in the multivariate logistic model. When a pair of continuous variables correlated significantly with *p* ≤ 0.05 (Spearman’s rank test), only one was tested by regression. Thus, ECP significantly correlated with eosinophil count regardless of the group, atopic (Spearman’s test, *p* = 0.05) or not (*p* = 0.02). Similar results were found for serum total IgE and specific anti-*Toxocara* IgE variables, either in atopic *(p* = 0.002) or nonatopic (*p* = 0.003) patients. Consequently, only eosinophil count and specific anti-*Toxocara* IgE were assessed by MRA. Environmental variables (see above) were not tested since MRA aimed only to assess the influence of atopy on the clinical and laboratory picture of toxocariasis.

Odds ratio estimates were adjusted for age and sex by logistic regression analysis; approximate 95% confidence limits were based on maximum likelihood estimates of logistic parameters.

## Results

Tables 1 and 2 display the results from the bivariate analysis of categorical or continuous demographic, clinical or laboratory variables. In the nonatopic group, female patients were significantly more present (Pearson’s χ^2^ test, *p* < 0.001). On average, patients in this group were significantly older than in the atopic group (Student’s test, *p* = 0.002). No significant difference was observed between nonatopic or atopic groups of patients concerning the distribution of clinical signs or symptoms. For laboratory parameters, only mean values of the eosinophil count or the dosage of ECP differed significantly (Mann-Whitney test, *p* = 0.02 for both). Interestingly, no significant difference was observed between groups concerning the mean values of serum total IgE or specific anti-*Toxocara* IgE.

Moreover, bivariate analysis of environmental parameters (listed above) did not find any significant difference between the two groups of patients.

The MRA results are shown in Table 3. These findings indicate that no clinical or laboratory covariate was found to be associated with atopy in our toxocariasis patients. Only age and sex were explanatory for the presence of an atopic status, but odds ratio values under 1.0 suggested an inverse correlation.

**Table 3:** Results of multivariate analysis of the association of atopy with variables found significant at *p ≤* 0.15.

Variable	*p*	Odds ratio	95% confidence interval
Age	0.009	0.97	[0.95 – 0.99]
Sex	0.003	0.26	[0.11 – 0.62]

## Discussion

Among 57 nonatopic patients (42 females and 15 males) and 49 atopic patients (20 females and 29 males) who had been diagnosed with active common/covert toxocariasis, MRA of demographic, clinical or laboratory parameters showed that the outcome variable “atopy” was explained only by age and sex (Table 3). This result indicates that our atopic patients were more often younger and of male sex than nonatopic patients. The presence of an elevated proportion of males among atopic patients has been well established [17, 33] and supports our MRA result. Conversely, sensitization to inhalant or food allergens and clinical expression of allergic diseases are recognized as increasing during the course of life [20, 40]. This point is not consistent with our findings.

In fact, the MRA result was due to an overrepresentation of females in the nonatopic group (sex ratio: 0.36). Moreover, these nonatopic female patients were on average significantly older than their atopic counterparts (Mann-Whitney *U* test, *p* = 0.036), whereas no significant difference was retrieved between the groups in the distribution of age values for males. This sex-ratio bias was surprising, since a meta-analysis of 250 seroprevalence surveys about toxocariasis demonstrated that male sex was a constant risk factor for this zoonosis [34]. According to other recognized features of toxocariasis epidemiology, the distribution of environmental parameters was tested *vs.* sex. The above-mentioned recruitment bias for nonatopic patients could not be attributed to occupation, number of pet dogs or cats at home, presence of a kitchen garden, or type of residence (urban, semi-urban or rural). Therefore, the action of a confounder that is not related to the epidemiology or pathophysiology of toxocariasis must be suspected. It would explain why nonatopic female patients presenting with toxocaral disease most frequently attended the clinic.

Bivariate analysis of various clinical variables followed by MRA MRA revealed that the clinical pictures in our patients were not dependent on atopy. First, the signs or symptoms observed during the course of common/covert toxocariasis are not specific [21, 24]. Therefore, it may be suspected that other disease conditions could have caused some clinical manifestations. For example, a prospective study on the causes of pruritus *sine materia* in 95 patients found that toxocariasis accounted for only 8.4% of the cases [2]. Second, in the wake of the well-known “hygiene hypothesis”, a theory arose about the relationship between helminthiases and atopy. Briefly, the intense Th2-skewed immunological response that is a hallmark of these infections appears to protect the host from developing allergic symptoms [41]. Further investigations suggested that during the course of chronic helminthiases, immunoregulation was boosted due to certain mechanisms, such as the development of regulatory T and B cells inducing immune hyporesponsiveness [10, 35]. Whether patients enrolled in the present study could be considered as having chronic helminthiasis was therefore a crucial point. Regardless of their status, atopic or not, they displayed a similar and long (approximately 6 months, Table 2) average time interval between onset of clinical manifestations accompanied by blood eosinophilia and attendance at the specialized clinic. Thus, the course of toxocaral disease observed in this study can be considered chronic.

Bivariate analysis of laboratory parameters showed a significantly increased level of blood eosinophils and ECP in nonatopic patients (Table 2). By MRA, blood eosinophilia was not considered dependent (Table 3). Epidemiology did not provide any explanation, since the bivariate analysis of environmental parameters (see above) did not reveal any correlation with the level of eosinophilia. Fundamental immunology may contribute to an explanatory hypothesis. During experimental helminth infections in animal models, eosinophils were found to accumulate in lymphoid organs [16]. Moreover, during the allergic process, these cells amass at sites of allergic inflammation [39]. Therefore, it could be hypothesized that the combination of both mechanisms tends to slightly lessen the number of circulating blood eosinophil cells in atopic toxocariasis patients. A further study including a greater number of patient records is needed to verify this hypothesis.

In both groups of patients, the level of serum total IgE was similarly elevated and was approximately 3-fold above the upper value of the normal range (Table 2). A substantial and sustained increase in this class of immunoglobulins in helminth-infected subjects was reported for the first time 50 years ago [18]. It was found to be a major laboratory abnormality during common toxocariasis [11]. The underlying mechanism remains poorly understood [9, 19] but does not seem to be modulated by the presence of the atopy genotype [32]. This production of high amounts of polyclonal IgE appears to cause saturation of mast cell Fcε receptors, thus supporting the claimed protective action of helminthiases against the onset of allergic diseases [14, 27].

The level of anti-TES-Ag IgE did not significantly differ between atopic and nonatopic patients and was correlated with the amount of serum total IgE (Table 2). MRA did not retain this variable as dependent on the atopy outcome variable. Therefore, production of anti-TES-Ag IgE appeared to be due largely to polyclonal immunostimulation and not to a specific reaction related to the presence of the atopy genotype.

Our results are partially consistent with the findings of Gonzalez-Quintela et al.’s [13] prospective cross-sectional study. These authors investigated 463 Spanish patients in whom they performed toxocariasis serology, skin prick test (SPT), and detection in serum of IgE specific for common inhalant allergens. A questionnaire inquired about the presence of respiratory symptoms. An MRA of demographic, environmental, clinical and laboratory variables was carried out using the result of toxocariasis serology as the outcome variable. SPT and detection of specific IgE were found to correlate, so only SPT results were tested by regression. Exposure to toxocaral infection was associated with an increase in both serum IgE levels and eosinophil counts in SPT-negative individuals. An opposite trend was observed in SPT-positive patients. No difference was observed concerning respiratory symptoms.

This finding of an increased level of eosinophilia in SPT-negative (nonatopic) subjects aligns with our results from bivariate analysis but not from MRA. The logistic model in the Spanish study, where exposure to toxocariasis was the outcome variable, differed from ours, where atopy was the variable to explain. Nonetheless, our hypothesis about the pathophysiology of this increased eosinophilia level in nonatopic patients may apply to both studies.

In the Spanish survey, an increased concentration of total IgE was also found in SPT-negative subjects exposed to toxocariasis, whereas our study showed that the level of this class of immunoglobulin was similar between both groups of patients. Importantly, exposure to an infectious agent, such as determined by a positive serology result, is a paradigm that differs from the presence of the related disease. The finding of specific antibodies in serum may correspond to an active infectious process or to past – repeated or not – self-cured infection. Using exposure to toxocariasis as an outcome variable thus elicited heterogeneous recruitment that may induce biases related to the pathophysiology of the zoonosis.

In conclusion, our study suggests that atopy does not influence the clinical and laboratory picture of patients with toxocaral disease, but does not provide any clarification about the reported increased seroprevalence of toxocariasis in atopic subjects. However, the presence in our study of a recruitment bias of unknown origin, affecting the sex ratio in the group of nonatopic patients, requires that these results be considered with caution.

## Conflict of interest

The authors declare that they have no competing interests.
